# Efficient Repopulation of Genetically Derived Rho Zero Cells with Exogenous Mitochondria

**DOI:** 10.1371/journal.pone.0073207

**Published:** 2013-09-03

**Authors:** Sandra Heller, Susanna Schubert, Mario Krehan, Ingo Schäfer, Martina Seibel, Dominga Latorre, Gaetano Villani, Peter Seibel

**Affiliations:** 1 Molecular Cell Therapy, Center for Biotechnology and Biomedicine (BBZ), Universität Leipzig, Leipzig, Germany; 2 RhoZero Technologies, Uettingen, Germany; 3 Department of Basic Medical Sciences, Neurosciences and Sense Organs, School of Medicine, University of Bari, Bari, Italy; University of Texas Health Science Center at San Antonio, United States of America

## Abstract

Mitochondria are involved in a variety of cellular biochemical pathways among which the ATP production by oxidative phosphorylation (OXPHOS) represents the most important function of the organelle. Since mitochondria contain their own genome encoding subunits of the OXPHOS apparatus, mtDNA mutations can cause different mitochondrial diseases. The impact of these mutations can be characterized by the trans-mitochondrial cybrid technique based on mtDNA-depleted cells (ρ^0^) as acceptors of exogenous mitochondria. The aim of the present work was to compare ρ^0^ cells obtained by long term ethidium bromide treatment and by a mitochondrial targeted restriction endonuclease, respectively, as mitochondrial acceptors for trans-mitochondrial cybrid generation. Fusion cells have mitochondrial respiratory functions comparable to their parental wild type cells, regardless the strategy utilized to obtain the ρ^0^ acceptor cells. Therefore, the newly developed enzymatic strategy for mtDNA depletion is a more convenient and suitable tool for a broader range of applications.

## Introduction

Mitochondria are the center of a variety of biochemical pathways that are involved in an ever increasing number of cellular physiological processes. Among them, the ATP synthesis through the oxidative phosphorylation (OXPHOS) represents the most important and the best characterized task that makes this organelle the powerhouse of aerobic eukaryotic cells [1], [2]. Mitochondria possess their own genome that encodes two rRNAs (12S and 16S subunit) and 22 tRNAs as major components of the translation system as well as 13 subunits of the OXPHOS apparatus [3].

Therefore, impairment of OXPHOS by mitochondrial DNA (mtDNA) mutations can cause mitochondrial diseases with a broad spectrum of clinical manifestations, for example blindness, deafness, dementia or cardiac failure [2]. Because of a possible heteroplasmic distribution of mtDNA mutations, threshold effects arising from different mutational loads can be observed strongly depending on the level of oxidative metabolism as well as on intrinsic OXPHOS properties of the affected tissues. Common diseases triggered by mtDNA mutations are Leber’s hereditary optic neuropathy (LHON) or neuropathy, ataxia and retinitis pigmentosa (NARP) resulting from an amino acid replacement or myoclonic epilepsy and ragged-red fiber disease (MERRF) and mitochondrial encephalomyopathy, lactic acidosis and stroke-like symptoms (MELAS), where alterations of a tRNA gene cause the disease [4]–[7].

The detailed characterization of the functional impact of the above-named pathogenic mtDNA mutations has been facilitated by the trans-mitochondrial cybrid technique based on the production and utilization of mtDNA-depleted cells (ρ^0^) as acceptors of exogenous mitochondria [8].

The original method to generate ρ^0^ cells was based on the long term treatment with DNA intercalating chemicals like ethidium bromide (EtBr) [9]. Disadvantages of this method are the long time exposure and the potential mutagenic side effect of the drug on nuclear DNA [10]. Therefore, we have developed a new method taking advantage of a mitochondrial targeted restriction endonuclease that destroys mtDNA within a few days [11].

The ρ^0^ cells possess unique growth requirements. Without an active respiratory chain these cells are dependent on uridine [12]. In addition, human ρ^0^ cells need pyruvate for normal growth possibly to oxidize the excess of cytoplasmic NADH via lactate dehydrogenase [13].

The aim of the present work was to compare ρ^0^ cells derived from the two described methods as mitochondrial acceptors for trans-mitochondrial cybrid generation in order to verify if the enzymatic strategy for mtDNA depletion is suitable for a broader range of applications. Therefore, the two ρ^0^ cell lines were fused to wild type cytoplasts from their parental cell line and the mitochondrial bioenergetic properties of the resulting cybrids were analyzed.

## Materials and Methods

### Cell Culture

Human osteosarcoma cells 143B.TK^-^ (ATCC CRL-8303) were cultured under standard conditions (37°C, 5% CO_2_) in Dulbecco’s Modified Eagle’s Medium (DMEM, high glucose) supplemented with 10% fetal calf serum (FCS), 100 µg/ml bromodeoxyuridine (BrdU) and 50 µg/ml uridine. Human prostate adenocarcinoma cells PC-3 (ATCC CRL-1435) were cultured under standard conditions in 45% Roswell Park Memorial Institute 1640 Medium (RPMI 1640) and 45% Ham’s F12 Medium supplemented with 10% FCS, 2 mg/ml glucose and 50 µg/ml uridine. Selective media without uridine was prepared utilizing 10% dialyzed FCS. The ρ^0^ cells were generated by cultivation of parental cells with low doses of ethidium bromide (as described in [13]).

Alternatively, transient transfections of PC-3 with mitochondrial targeted restriction endonuclease [11] were performed using *Trans*IT®-LT1 transfection reagent (Mirus Bio Corporation, Madison, USA) according to manufacturer’s conditions. ρ^0^ clone PC-3 9B4 isolated after cell transfection was aliquoted and metabolically tested for uridine auxotrophy in the selective medium described above.

Parental cell lines were stably transfected with a plasmid coding mitochondrial targeted DsRed (MTS-DsRed) using geneticin selection. Cell culture reagents were obtained from Biochrom AG (Berlin, Germany).

### Cell Fusion

Enucleation of parental cells stably expressing MTS-DsRed was performed utilizing the Ficoll gradient technique (as described by [14]) modified by King and Attardi [15]. Parental cells (0.4–1×10^7^) were layered on a discontinuous Ficoll gradient using 12.5%, 15%, 16%, 17% and 25% Ficoll (Sigma-Aldrich GmbH, Taufkirchen, Germany) containing 1 mg/ml cytochalasin B (Sigma-Aldrich GmbH, Taufkirchen, Germany) and were centrifuged at 96760×g for 1 h at 37°C. Recovered cytoplasts of parental cells were fused for 60 s in polyethylene glycol solution (40%, PEG 1500, AppliChem GmbH, Darmstadt, Germany) to different resuspended ρ^0^ cells generated by ethidium bromide treatment and transfection at the same ratio of acceptor to cytoplast donor cells. Cell fusion was monitored by confocal microscopy. After cell fusion, plated cells were maintained for 24 h in media containing uridine and then cultivated in selective media. For each fusion cell line approximately ten cell clones with restored mitochondrial respiration were obtained after cell culture in selective media and cultured as a heterogeneous cell population.

The term PC-3 fusion cells refers to PC-3 ρ^0^ cells fused with cytoplasts of PC-3 MTS-DsRed (F 1–3) and PC-3 MTS-EGFP ρ^0^ cells (with stable expression of mitochondrial targeted EGFP) fused with cytoplasts of PC-3 MTS-DsRed (F A) or cytoplasts of 143B.TK^-^ MTS-DsRed (F B) (see Table 1).

**Table 1:** Nomenclature of fusion cell lines.

Cell line	Parental cell line	Treatment for mtDNA depletion	Stable transfection	Cytoplast donor cell line
WT	PC-3	–	**–**	–
WT	PC-3	–	MTS-DsRed	–
WT	143B.TK^−^	–	MTS-DsRed	–
PC-3 EtBr F 1	PC-3	EtBr	–	PC-3 MTS-DsRed
PC-3 EtBr F 2	PC-3	EtBr	–	PC-3 MTS-DsRed
PC-3 EtBr F 3	PC-3	EtBr	–	PC-3 MTS-DsRed
PC-3 9B4 F 1	PC-3	transfection	–	PC-3 MTS-DsRed
PC-3 9B4 F 2	PC-3	transfection	–	PC-3 MTS-DsRed
PC-3 9B4 F 3	PC-3	transfection	–	PC-3 MTS-DsRed
PC-3 9B4 F A	PC-3	transfection	MTS-EGFP	PC-3 MTS-DsRed
PC-3 9B4 F B	PC-3	transfection	MTS-EGFP	143B.TK^-^ MTS-DsRed

After maintaining fused cells for several weeks in cell culture, growth analysis in media with and without uridine was performed. Cell number was quantified over a period of six days and cell doubling time was calculated from exponential growth curve.

### Confocal Microscopy

Cells were plated on glass bottom dishes (MatTek Corporation, Ashland, USA) after cell fusion and analyzed with the inverted confocal laser scanning microscope TCS SP5 (Leica Microsystems GmbH, Wetzlar, Germany) after 24 h of cultivation. A sequential scanning mode was used to avoid a cross talk in excitation of multiple stained compounds. Images were acquired with photo multipliers and micrographs were processed with the software Leica Application Suite Advanced Fluorescence 2.6.0 and Adobe Photoshop CS.

### DNA Analysis (PCR and Real-Time PCR)

The loss of endogenous mitochondrial DNA (mtDNA) and hence the ρ^0^ state of cells was controlled by PCR. Therefore, genomic DNA of parental and ρ^0^ cells was isolated and the D-Loop region of mtDNA (348-For 5′-CCAAACCCCAAAAACAAAGAA-3′ and 783-Rev 5′-TTTGAGCTGCATTGCTGCGTG-3′) and 18S rDNA of nuclear DNA (18S-0899-For 5′-TCGGAACTGAGGCCATGATTA-3′ and 18S-1127-Rev 5′-GCGGGTCATGGGAATAACG-3′) were amplified using PCR.

Gene integration and transcription of EcoRI coding sequence after transient transfection was verified with following primers: EcoRIR-001-For 5′-CATGGACGAGCTGTACAAGATGTCTAATAAAAAAC-3′, EcoRIR-220-For 5′-GACCCTGATCTTGGCGGTACTTTATTTG-3′, EcoRIR-392-For 5′-GAGGAGATCAAGATTTAATGGCTGCTG-3′, EcoRIR-653-Rev 5′-CATAGATTACTATTTATAGGCATTCCATAATTAGCTGC-3′, EcoRIR-819-Rev 5′-CTGTTCAAACAAGTCACGCC-3′ and EcoRIR-834-Rev 5′-GGCCAAATCACTTAGATGTAAGCTGTTCAAAC-3′. Primer combinations for amplification of EcoRI gene sections are listed in Table 2.

**Table 2:** Primer combinations for amplification of EcoRI gene sequence sections.

Forward primer	Reverse primer	Amplified fragment	Primer pair
EcoRIR-001-For	EcoRIR-834-Rev	860 bp	A
EcoRIR-001-For	EcoRIR-819-Rev	838 bp	B
EcoRIR-220-For	EcoRIR-653-Rev	434 bp	C
EcoRIR-392-For	EcoRIR-834-Rev	450 bp	D
EcoRIR-392-For	EcoRIR-653-Rev	262 bp	E

Gene integration of DsRed into nuclear genome was studied by primers DsRed-001-For 5′-ATGGTGCGCTCCTCCAAGAACG-3′ and DsRed-681-Rev 5′-CTACAGGAACAGGTGGTGGCGG-3′.

Relative quantification of mtDNA and nuclear DNA was analyzed utilizing a 7500 Real Time PCR System (Applied Biosystems, Foster City, USA) and SYBR® Green as double-strand DNA-specific binding dye (Eurogentec, Seraing, Belgium), according to instructions of manufacturer. Primers amplifying the 18S rDNA (18S-1036-For 5′-AGTCGGAGGTTCGAAGACGAT-3′ and 18S-1127-Rev 5′-GCGGGTCATGGGAATAACG-3′, nuclear) and ND5 gene (12574-For 5′-TTCAAACTAGACTACTTCTCCATAATATTCATC-3′ and 12674-Rev 5′-TTGGGTCTGAGTTTATATATCACAGTGA-3′, mitochondrial) were used for the relative evaluation of mtDNA compared to nuclear DNA of parental and fused cell lines according to the ΔCt method.

### RNA Analysis

DNase-treated total RNA was reverse transcribed with Oligo-dT_18_ primers and expression of β-actin (control) and EcoRI were studied by PCR using primers ACTB-0187-For 5′-GGCATCCTCACCCTGAAGTA-3′ and ACTB-0732-Rev 5′-GTCAGGCAGCTCGTAGCTCT-3′ for β-actin and EcoRI primer pairs A and E for EcoRI (see Table 2). Control cells were transfected with mitochondrial targeted restriction endonuclease and harvested 48 h post transfection.

### Measurements of Endogenous Respiration and (Ascorbate+TMPD)-dependent Respiration in Intact Cells

Exponentially growing cells with a medium change the day before measurement were trypsinized and harvested by centrifugation (180×g, 5 min). Cells were resuspended at a density of 2–4×10^6^ cells per ml in Tris-based, Mg^2+^- and Ca^2+^-deficient (TD) buffer (25 mM Tris-HCl pH 7.4 at 25°C, 0.137 M NaCl, 5 mM KCl, 0.7 mM Na_2_HPO_4_) at 37°C. Measurements were performed in a temperature-controlled chamber containing a stirring bar, connected to a circulating water bath at 37°C and a Clark type oxygen electrode (Hansatech Instruments, Pentney, England). Oxygen consumption was monitored directly and after serial addition of 2,4-dinitrophenol (DNP at 30 µM), antimycin A (13 nM), *N,N,N’,N’*-tetramethyl-*p*-phenylenediamine (TMPD) and ascorbate (0.4 mM and 10 mM) and potassium cyanide (KCN at 2 mM), respectively [16], [17].

### Biochemical Assays of Respiratory Chain Complex Activity

The enzyme activity of different respiratory chain complexes was measured in lysates from digitonin-permeabilized cells as previously described by Seibel *et al.* [18]. Cells were detached from culture dishes by trypsinization and cell pellet was resuspended in buffer A (30 mM Tris-HCl pH 7.4 at 25°C, 3 mM MgCl_2_, 0.5 mM Na-EDTA, 40 mM KCl, 5 mM KH_2_PO_4_, 75 mM saccharose). Cell suspension was treated for 2 min with digitonin (40 µg per 10^6^ cells) and inactivated with buffer A containing 0.35% BSA. The pellet was then resuspended in hypotonic buffer containing protease inhibitor (Roche Applied Science, Mannheim, Germany) and subjected to three rounds of freeze/thaw prior to the enzymatic assay. Spectrophotometric assays of complex I, IV and citrate synthase were carried out with a Beckman DU800 spectrophotometer (Beckman Coulter, Krefeld, Germany) equipped with a rapid-mixing apparatus as described previously [18].

### EcoRI Activity Analysis

PC-3 wild type, PC-3 ρ^0^ 9B4 and PC-3 transfected with mitochondrial targeted restriction endonuclease (48 h post transfection) were collected and resuspended in potassium phosphate buffer (10 mM, pH 7.4) at 2.5×10^6^ cells per ml. Samples were homogenized by passing it through a syringe fitted with a 26-gauge needle. Cell lysate with 0.15% Triton X-100 containing protease inhibitor (Roche Applied Science, Mannheim, Germany) was incubated at a final concentration of approximately 2.5×10^5^ cells per ml with linear DNA fragment in EcoRI restriction buffer (50 mM Tris-HCl, pH 7.4 at 37°C, 10 mM MgCl_2_, 100 mM NaCl, 0.02% Triton X-100, 0.1 mg/ml BSA) at 37°C.

### Statistical Analysis

Data are presented as mean ± standard deviation. An unpaired two-sample *t*-test was performed to analyze the statistical significant differences using software SigmaPlot, version 12.0. Differences were considered significant at P<0.05.

## Results and Discussion

**Figure 1:**
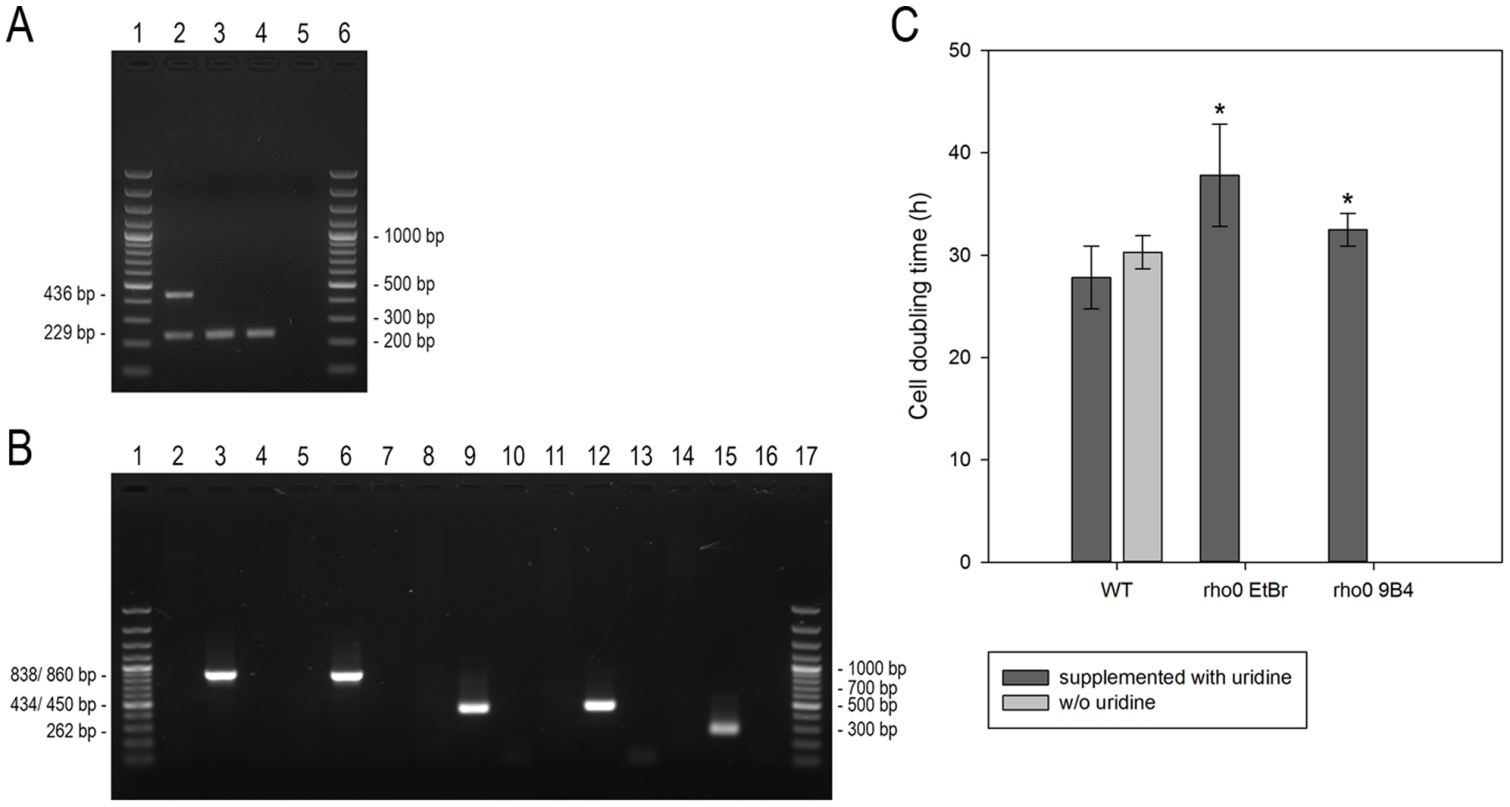
Characterization of ρ^0^ cells. A Multiplex PCR analysis of mitochondrial DNA. The nuclear and mitochondrial genes (18S rDNA: 229 bp and D-Loop: 436 bp, respectively) were coamplified and visualized by gel electrophoresis. Agarose gel (1.5%), lane 1 and 6: GeneRuler™ 100 bp plus DNA Ladder, lane 2: PC-3 wild type, lane 3: PC-3 ρ^0^ EtBr, lane 4: PC-3 ρ^0^ 9B4, lane 5: no template. **B** PCR analysis of EcoRI gene sequence in PC-3 ρ^0^ 9B4 cells. Different EcoRI gene sequences were amplified by PCR from PC-3 ρ^0^ 9B4 genomic DNA utilizing the primer pairs listed in Table 2. Agarose gel (1.5%), lane 1 and 17: GeneRuler™ 100 bp Plus DNA Ladder, lane 2–4: PC-3 ρ^0^ 9B4, vector DNA (encoding mitochondrial targeted restriction endonuclease, 500 pg) and no template, primer pair A, lane 5–7: primer pair B, lane 8–10: primer pair C, lane 11–13: primer pair D, lane 14–16: primer pair E. **C** Cell growth analysis of PC-3 cells. Calculation of doubling time of PC-3 cells in media with uridine (dark grey bars) and without uridine (light grey bars). Total cell number was measured every 24 h over a period of six days and cell doubling was estimated using exponential regression. The presented data are means ± SD from four independent experiments. *P<0.05, **P<0.01, ***P<0.001.

To study the mtDNA depletion process, PC-3 cells were treated with ethidium bromide (PC-3 ρ^0^ EtBr) or transfected with a targeted restriction endonuclease expressing construct (PC-3 ρ^0^ 9B4), respectively, to achieve complete depletion of mtDNA. The ρ^0^ state was confirmed both by genetic (PCR) and metabolic testing (uridine auxotrophy). As shown in Fig. 1 A, wild type cells exhibit both nuclear and mitochondrial PCR-amplified DNA markers (lane 2), whereas ρ^0^ cells completely lack the mitochondrial D-loop PCR product (lane 3 and 4). To verify the uridine auxotrophy, cell growth of both ρ^0^ cell lines was analyzed. The results are displayed in Fig. 1 C where the slightly higher doubling time of the same lines as compared with the parental cells can be also noted.

One of the possible problems arising from the use of the vector-based strategy to obtain the ρ^0^ cells could be the integration of the EcoRI gene in the nuclear genome that would lead to degradation of any newly introduced mtDNA.

To see whether this side effect takes place, we studied EcoRI gene sequence integration. Fig. 1 B shows that EcoRI sequences could be amplified only in vector DNA containing samples (lane 3, 6, 9, 12, 15), but not in the genomic DNA from PC-3 ρ^0^ 9B4. In addition, expression and enzyme activity of EcoRI was tested in PC-3 ρ^0^ 9B4 to exclude any negative effect on mtDNA complementation after cell fusion. EcoRI transcripts and enzyme activity could only be demonstrated for PC-3 48 h post transfection, but not in wild type and ρ^0^ cells (Fig. S1).

As a result of the experiments, both the ρ^0^ cell lines could be fused with cytoplasts from wild type cells for mtDNA complementation.

**Figure 2:**
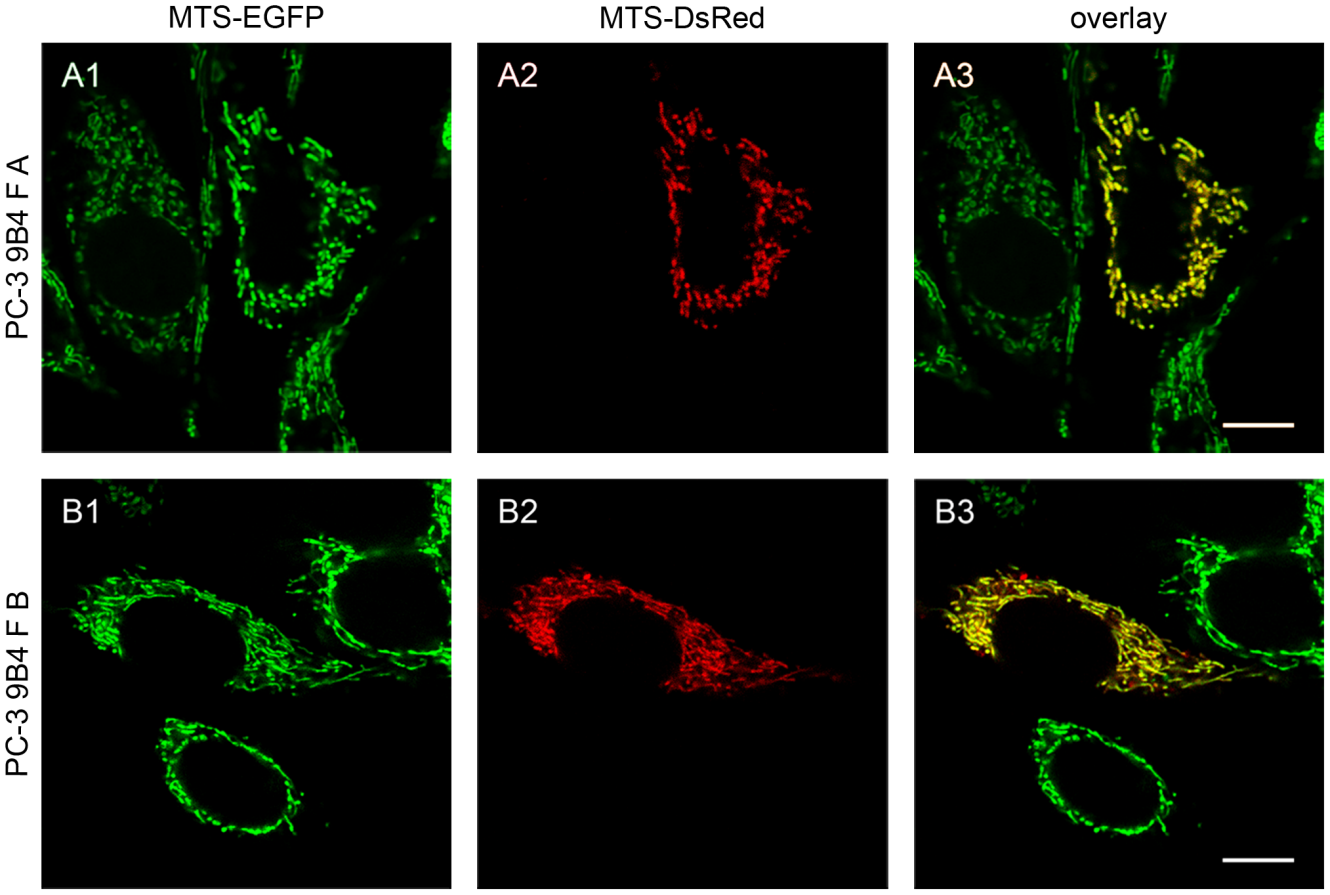
Confocal images of ρ^0^ cells fused with cytoplasts of wild type cells. PC-3 ρ^0^ 9B4 cells stably expressing mitochondrial targeted EGFP (MTS-EGFP) were fused with cytoplasts of PC-3 (panel A) or 143B.TK^-^ (panel B) stably expressing mitochondrial targeted DsRed (MTS-DsRed) and were analyzed after 24 h by confocal laser scanning microscopy. Scale bars correspond to 10 µm.

The classical trans-mitochondrial cybrid technique is based on the use of nuclear markers in the ρ^0^ cell line for the negative selection of mitochondrial donor cells that have not been successfully enucleated and could, therefore, represent a contamination of non-cybrid cells. In the case of wild type parental cytoplasts fused with their corresponding ρ^0^ cells this selection strategy cannot be utilized. For this reason we decided to fuse PC-3 ρ^0^ cells stably expressing a mitochondrial targeted EGFP with cytoplasts containing red fluorescent mitochondria from PC-3 (F A) as well as from a different mtDNA donor cell line (143B.TK^-^, F B (see Table 1)) and monitored them by confocal microscopy (Fig. 2). After 24 h fusion, we could detect ρ^0^ cells with green fluorescent mitochondria (Fig. 2, A1 and B1) as well as fused cells also exhibiting MTS-DsRed from cytoplast mitochondria (Fig. 2, A2 and B2) throughout the complete mitochondrial network (Fig. 2, A3 and B3). The mitochondrial red fluorescent staining was completely lost in nearly all cells after two weeks. Additionally, PC-3 ρ^0^ cells were fused with cytoplasts from a PC-3 donor cell line stably expressing MTS-DsRed (F 1–3, see Table 1). It must be noted that all the described fusion cell lines arose from multiple cell clones pooled together after the fusion event.

**Figure 3:**
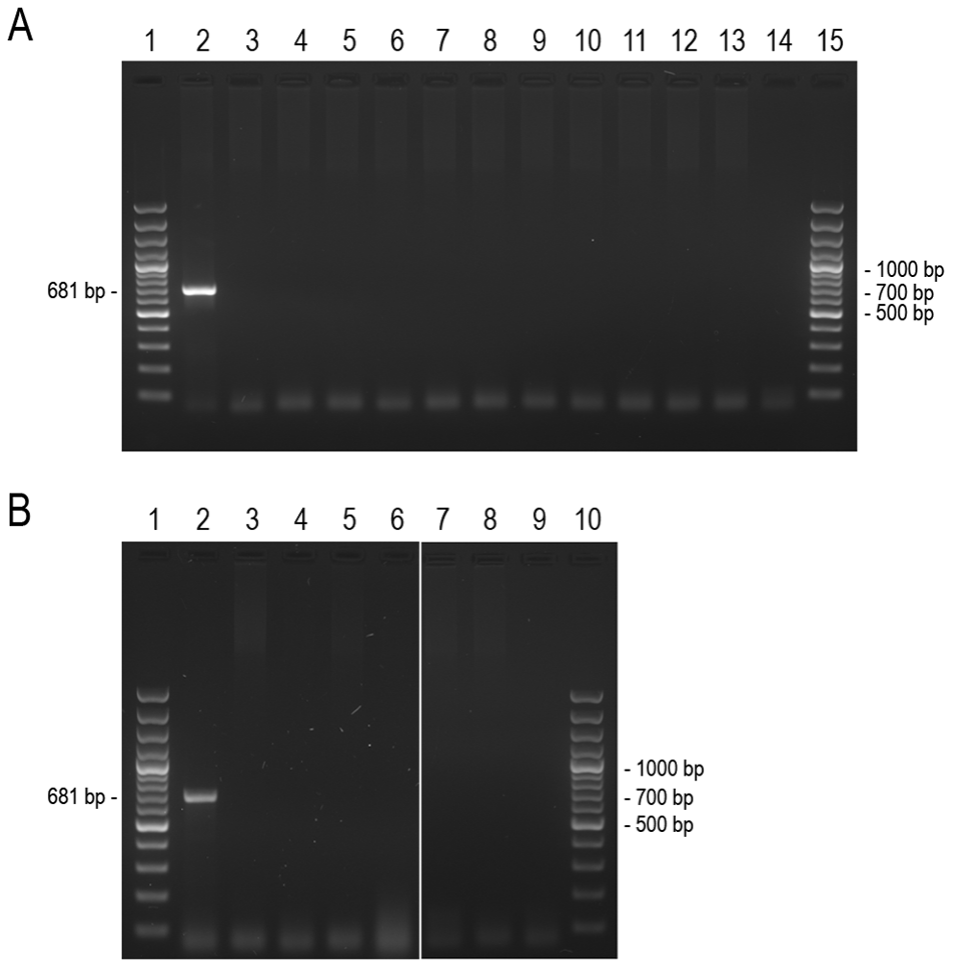
PCR analysis of MTS-DsRed gene sequence in nuclear genome. A 681 bp fragment of the DsRed gene was amplified by PCR as described in the experimental procedures. **A** Agarose gel (1.5%), lane 1 and 15: GeneRuler™ 100 bp plus DNA Ladder, lane 2: PC-3 MTS-DsRed, lane 3: EtBr F 1, lane 4: EtBr F 2, lane 5∶9B4 F 1, lane 6∶9B4 F 2, lane 7: EtBr F 4, lane 8: EtBr F 3, lane 9∶9B4 F 5, lane 10∶9B4 F 4, lane 11∶9B4 F 3, lane 12: PC-3 ρ^0^ EtBr, lane 13: PC-3 ρ^0^ 9B4, lane 14: no template control. **B** Agarose gel (1.5%), lane 1 and 10: GeneRuler™ 100 bp plus DNA Ladder, lane 2∶143B.TK^-^ MTS-DsRed, lane 3: PC-3 MTS-EGFP ρ^0^ EtBr, lane 4: PC-3 MTS-EGFP ρ^0^ 9B4, lane 5: EtBr F B, lane 6∶9B4 F B, lane 7: EtBr F A, lane 8∶9B4 F A lane 9: no template control.

In addition, genomic DNAs from all fusion cell lines were tested for the integrated DsRed gene sequence. As shown in Fig. 3 both ρ^0^ acceptor cells and fusion cell lines do not have any contamination with MTS-DsRed gene amplification product (Fig. 3 A: lane 3–13, Fig. 3 B: lane 3–8).

It is worth noticing that the use of ρ^0^ cells expressing mitochondrial fluorescent protein markers coupled to fluorescence-activated cell sorting could represent an efficient and faster alternative protocol for the selection of trans-mitochondrial cybrids.

**Figure 4:**
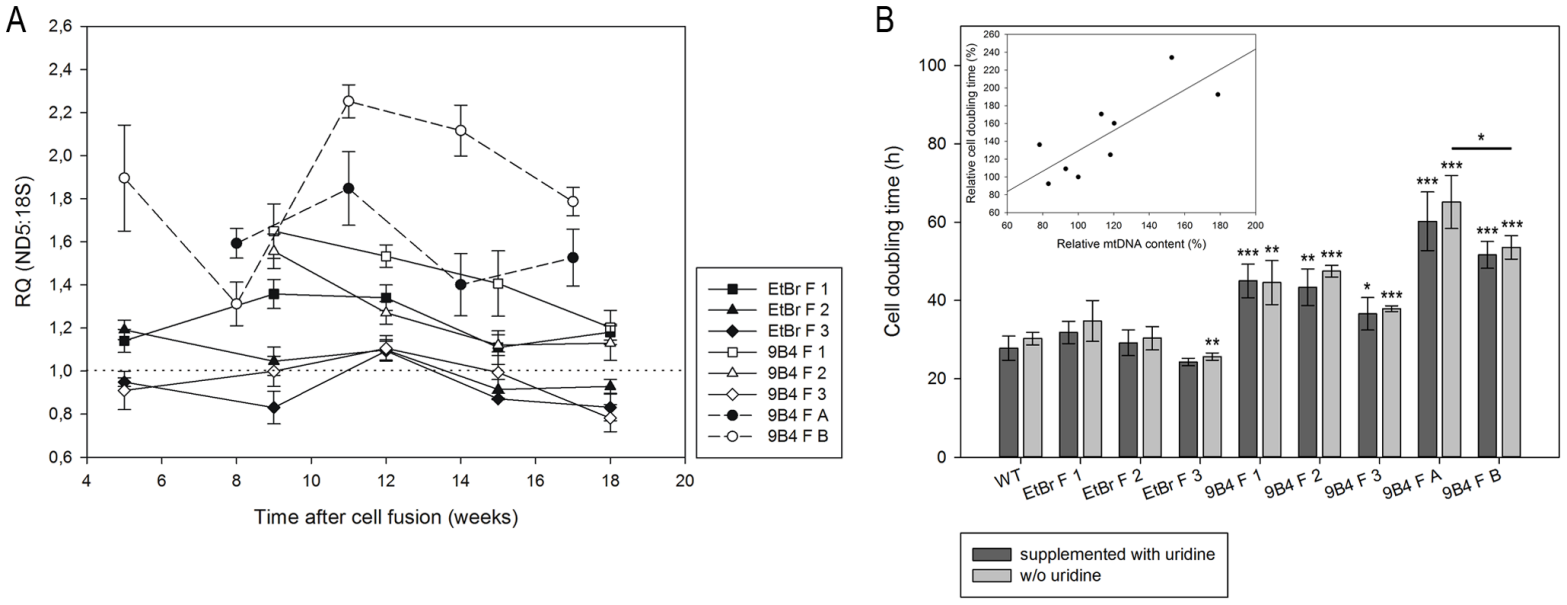
Relative quantification of mtDNA and cell growth analysis of PC-3 fusion cells. A Analysis of mtDNA relative to nuclear DNA over a period of 18 weeks after cell fusion of PC-3 ρ^0^ cells and cytoplasts of PC-3 MTS-DsRed (F 1–3, solid lines) or PC-3 MTS-EGFP ρ^0^ cells and cytoplasts of PC-3 MTS-DsRed (F A) and cytoplasts of 143B.TK^-^ MTS-DsRed (F B) (dashed lines) using a primer set for amplification of nuclear (18S rDNA) and mitochondrial (ND5 gene) DNA. Data were normalized to measurements of untreated wild type cells (dotted line). Data represent means ± SD obtained in three determinations. **B** Calculation of doubling time of PC-3 cells in media with uridine (dark grey bars) and without uridine (light grey bars). Total cell number was measured every 24 h over a period of six days and cell doubling was estimated using exponential regression. The presented data are means ± SD obtained in four experiments. Significance levels between wild type and the different fusion cell lines or if designated between two fusion cell lines were indicated. *P<0.05, **P<0.01, ***P<0.001. *B Inset* Comparison of relative mtDNA content and cell doubling time. MtDNA content and cell doubling time of PC-3 fusion cells cultured without uridine (close symbols) were normalized to wild type cells and a linear regression was performed (solid line).

To compare the efficiency of mtDNA repopulation in the different cybrid lines, the mtDNA was quantified by Real-Time PCR over a period of up to 18 weeks after the fusion (Fig. 4 A). Fusion cells (F 1–3, solid lines) exhibit a slightly increased amount of mtDNA shortly after the fusion event, but 18 weeks after cell fusion a decline to a level comparable to wild type cells could be observed (RQ = 1, dotted line). No significative difference between cells derived from ethidium bromide or vector-treated ρ^0^ cells could be detected. In contrast, stable MTS-EGFP transfected fusion cells with different mtDNA donors (F A-B, dashed lines) display an elevated level of mtDNA (Fig. 4 A). This increase in mtDNA level is independent of the donor when comparing the respective fusion cells complemented with PC-3 (F A) and 143B.TK^-^ mtDNA (F B), respectively, and could indicate an aspecific effect upon the transfection procedure. The analysis could show that ρ^0^ acceptor cells were successfully repopulated with mtDNA.

Furthermore, cell growth rate analysis (Fig. 4 B) shows that the doubling time measured in media with and without uridine is not significantly different in the fused cell lines. On the other hand, while EtBr fusion cells (EtBr F 1–3) exhibit a doubling time comparable with wild type cells, 9B4 fusion cells show a significant slower growth rate (9B4 F 1–3). Besides, 9B4 ρ^0^ acceptor cells fused with PC-3 and 143B.TK^-^ cytoplasts (9B4 F A and B) possess also a longer cell doubling time.

Interestingly, we could find a quite linear correlation between the amount of mtDNA and the cell doubling time of cells cultured in media without uridine (inset in Fig. 4 B). The same linear correlation was observed for cells cultured in uridine-supplemented media (data not shown). This could be possibly explained by the slow metabolic adaptation of the fusion cells to the reappearance of oxidative metabolism starting from the strictly anaerobic glycolytic metabolism of ρ^0^ cells, specifically characteristic of 9B4 fusion cells with a reduced growth rate.

**Figure 5:**
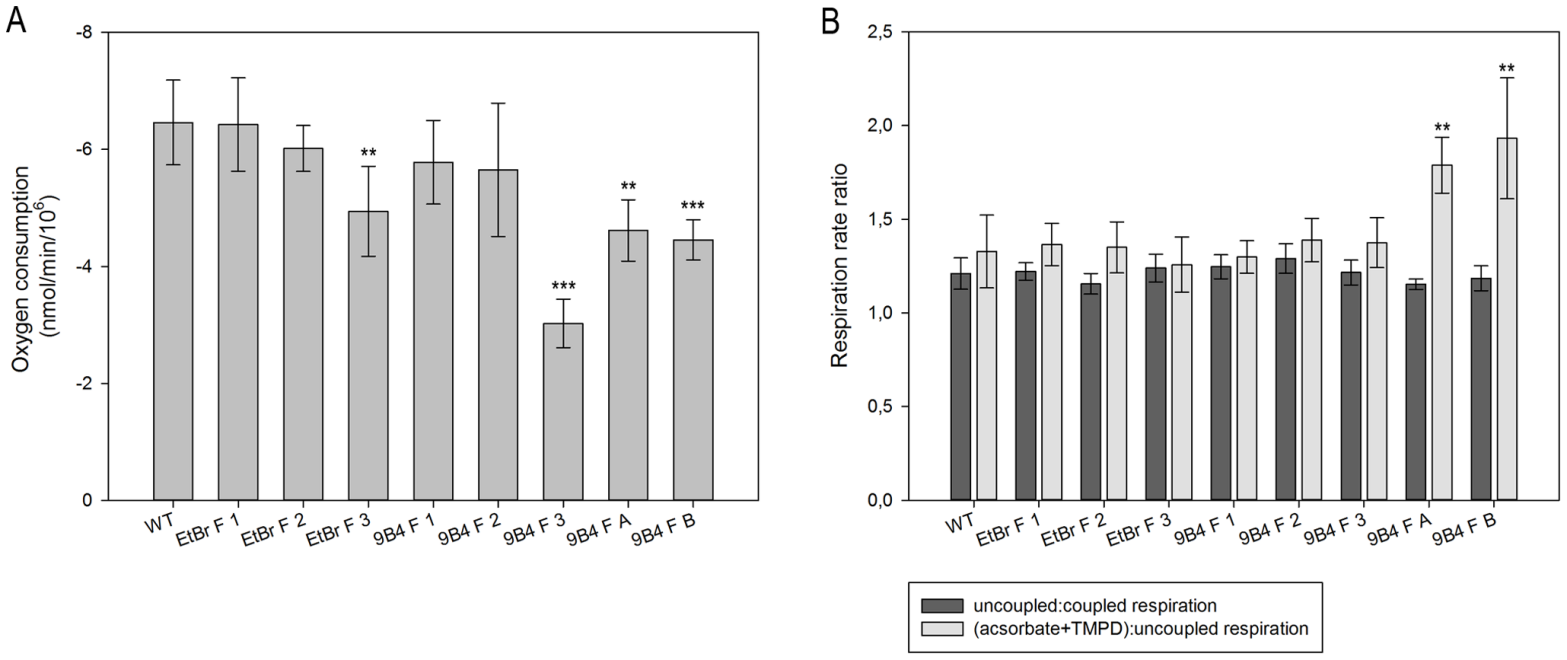
Polarographic respiration measurements of PC-3 fusion cells. **A** Endogenous oxygen consumption rate of intact PC-3 fusion cells was measured in TD buffer and normalized to cell number of the sample. **B** Relative oxygen consumption rates of PC-3 fusion cells were measured in TD buffer in absence and presence of the uncoupler DNP (dark grey bars). COX capacity of PC-3 fusion cells was measured as relative uncoupled respiration rate at 0.4 mM TMPD in antimycin-treated cells (light grey bars). The data represent means ± SD from 4–6 independent experiments. *P<0.05, **P<0.01, ***P<0.001.

To characterize the mitochondrial bioenergetic properties of fusion cells, a functional analysis was carried out by polarographic measurement of respiratory fluxes in intact cells. As shown in Fig. 5 A, fusion cells recover their basal respiratory activity to values that are comparable or slightly lower than those of wild type cells with the exception of 9B4 F 3 cells exhibiting a reduction of about 50% as compared to wild type cells. Therefore, a functional complementation of mtDNA was quantitatively achieved regardless of the applied mtDNA depletion strategy utilized for the acceptor cells. This was confirmed by the measurement of the DNP-uncoupled respiration rate that was assayed to exclude any differences in the control of the endogenous respiration by the steady-state mitochondrial membrane potential (Fig. 5 B, dark grey bars).

Additionally, the cytochrome c oxidase (COX) reserve capacity was assayed by a simplified approach [16], and also confirmed by KCN titration experiments (data not shown) as an intrinsic property of the cellular respiratory flux that can vary in different cell types [19] and/or as a result of mutations altering the relative proportion of the respiratory complexes [16]. As shown in Fig. 5 B (light grey bars), PC-3 fusion cells with the same mtDNA donor possess no alteration in the excess of COX capacity compared to wild type cells. In contrast, PC-3 9B4 F A and F B cells display an increase in COX capacity.

**Figure 6:**
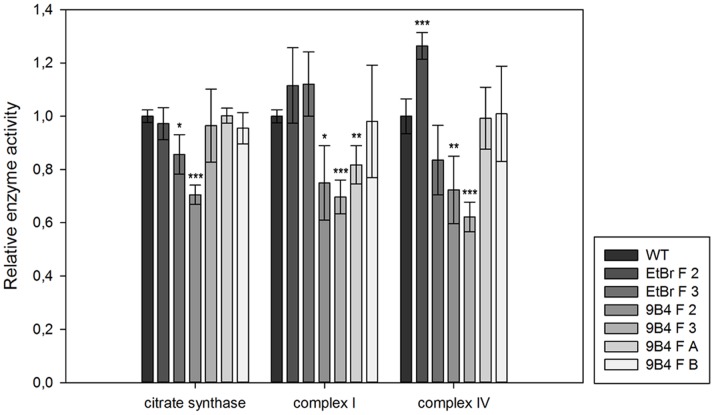
Activity of mitochondrial enzymes in PC-3 fusion cells. Enzyme activity of mitochondrial citrate synthase, respiratory complex I and IV was measured spectrophotometrically in total cell lysates and normalized to wild type. The data represent means ± SD from four independent experiments. *P<0.05, **P<0.01, ***P<0.001. Specific activity means of mitochondrial enzymes are shown in Table S1.

In addition to the *in vivo* respiration measurements, the enzyme activity of selected respiratory chain complexes and citrate synthase was measured on cell lysates (Fig. 6). All the cell lines recovered complex I and complex IV enzyme activities and the variations from the wild type values did not depend on the ρ^0^ acceptor cell types. PC-3 9B4 fusion cell lines with two different mtDNA donors display an enzyme activity comparable to wild type cells without significant differences between 9B4 F A and 9B4 F B. The same conclusions could be drawn when analyzing complex I and IV activity normalized to citrate synthase activity (Fig. S2), where respiratory complex activity is corrected for variations in protein amount used for the assay. A significant reduction of complex I and IV in 9B4 F 3 cells is in line with their reduced respiratory capacity (Fig. 5 A). Additionally, a significant increase of complex IV activity in EtBr F 2 could be observed.

## Conclusions

In conclusion, fusion cybrids have mitochondrial respiratory functions comparable to their parental wild type cells, regardless the strategy utilized to obtain the ρ^0^ acceptor cells. The differences of the fusion cells derived from the PC-3 9B4 ρ^0^ cells stably expressing mitochondrial targeted EGFP could be ascribed to a specific change occurred in the upstream clonal selection of the MTS-EGFP positive transformants. Therefore, in the light of the advantages so far evidentiated (see also ref. [11]), the vector-derived ρ^0^ cells represent a more convenient and suitable tool for the trans-mitochondrial cybrid technique and, consequently, for the detailed characterization of the phenotypic impact of mtDNA mutations.

## Supporting Information

Figure S1 **Expression and activity of EcoRI in PC-3 cells.** A PCR analysis of reverse transcribed total RNA. Expression of β-actin (control, 546 bp) and EcoRI (860 bp and 262 bp) was verified as described in the experimental procedures. Agarose gel (1.5%), lane 1, 9 and 18: GeneRuler™ 100 bp plus DNA Ladder, lane 2–8: β-actin primers, lane 10–13: EcoRI primer pair A, lane 14–17: EcoRI primer pair E, lane 2, 10 and 14: cDNA of PC-3 WT, lane 3, 11 and 15: cDNA of PC-3 ρ^0^ 9B4, lane 4, 12 and 16: cDNA of transfected PC-3, lane 5–7: RNA control of PC-3 WT, PC-3 ρ^0^ 9B4 and transfected PC-3, lane 8, 13 and 17: no template control. **B** Characterization of EcoRI activity in cell lysates through restriction of a DNA fragment containing an EcoRI recognition sequence. DNA fragment was incubated with cell lysates of PC-3 wild type, PC-3 ρ^0^ 9B4 and transiently transfected PC-3 cells for 30 min, 60 min and 120 min. Control restriction analysis was performed with purified restriction enzyme EcoRI for 30 min. Agarose gel (1.5%), lane 1 and 11: GeneRuler™ 100 bp plus DNA Ladder, lane 2–4: cell lysate of PC-3 WT, PC-3 ρ^0^ 9B4 and transfected PC-3, 30 min incubation, lane 5–7∶60 min incubation, lane 8–10∶120 min incubation, lane 12: untreated, lane 13: purified EcoRI.
(TIF)

Figure S2 **Relative activity of mitochondrial enzymes in PC-3 fusion cells.** Enzyme activity of respiratory complex I and IV was measured spectrophotometrically in total cell lysates and was normalized to citrate synthase activity as reference activity. The data shown as ratio of wild type cells represent means ± SD from four independent experiments. *P<0.05, **P<0.01, ***P<0.001. Specific activity means of mitochondrial enzymes are shown in Table S1.
(TIF)

Table S1 **Activity means of mitochondrial enzymes in PC-3 fusion cells.**
(DOC)
