# Supplementary figures and images for: Efficient Repopulation of Genetically Derived Rho Zero Cells with Exogenous Mitochondria
Source: PLoS One. 2013 Sep 3;8(9):e73207. doi: 10.1371/journal.pone.0073207 (PMC3760891; doi:10.1371/journal.pone.0073207)

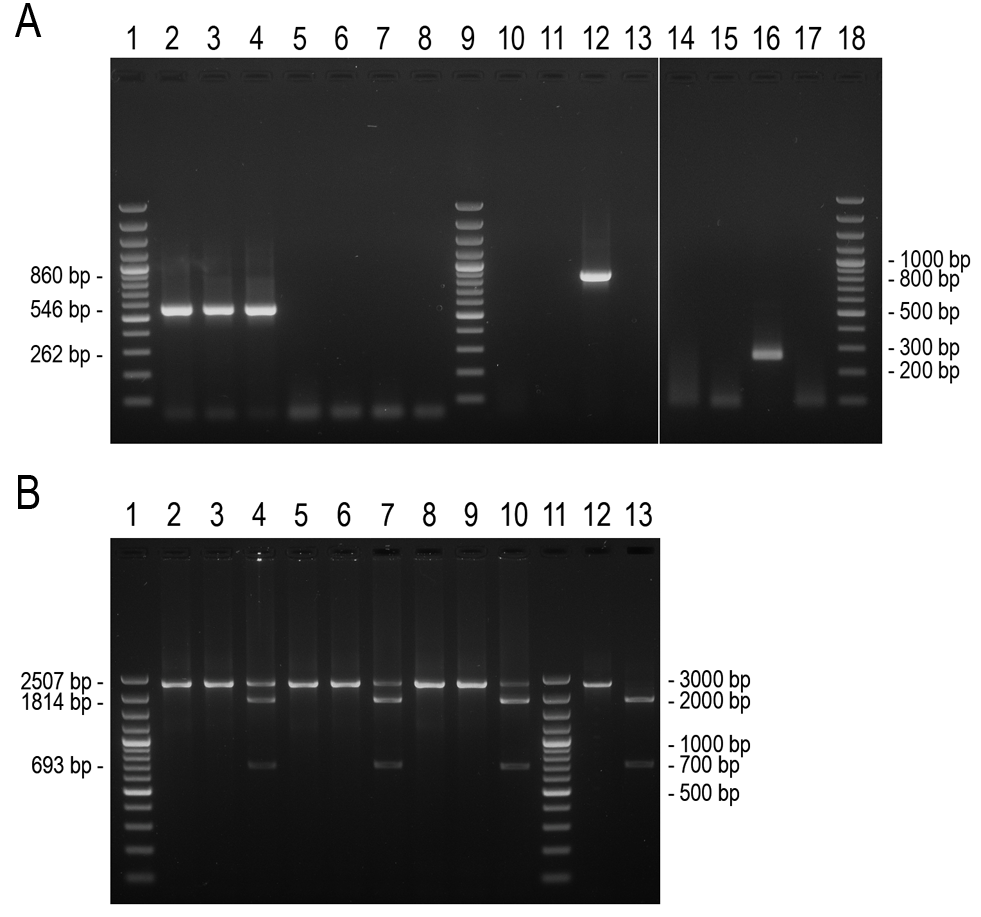

Supplement: Figure S1 — Expression and activity of EcoRI in PC-3 cells. A PCR analysis of reverse transcribed total RNA. Expression of β-actin (control, 546 bp) and EcoRI (860 bp and 262 bp) was verified as described in the experimental procedures. Agarose gel (1.5%), lane 1, 9 and 18: GeneRuler™ 100 bp plus DNA Ladder, lane 2–8: β-actin primers, lane 10–13: EcoRI primer pair A, lane 14–17: EcoRI primer pair E, lane 2, 10 and 14: cDNA of PC-3 WT, lane 3, 11 and 15: cDNA of PC-3 ρ0 9B4, lane 4, 12 and 16: cDNA of transfected PC-3, lane 5–7: RNA control of PC-3 WT, PC-3 ρ0 9B4 and transfected PC-3, lane 8, 13 and 17: no template control. B Characterization of EcoRI activity in cell lysates through restriction of a DNA fragment containing an EcoRI recognition sequence. DNA fragment was incubated with cell lysates of PC-3 wild type, PC-3 ρ0 9B4 and transiently transfected PC-3 cells for 30 min, 60 min and 120 min. Control restriction analysis was performed with purified restriction enzyme EcoRI for 30 min. Agarose gel (1.5%), lane 1 and 11: GeneRuler™ 100 bp plus DNA Ladder, lane 2–4: cell lysate of PC-3 WT, PC-3 ρ0 9B4 and transfected PC-3, 30 min incubation, lane 5–7∶60 min incubation, lane 8–10∶120 min incubation, lane 12: untreated, lane 13: purified EcoRI. (TIF) [file pone.0073207.s001.tif]

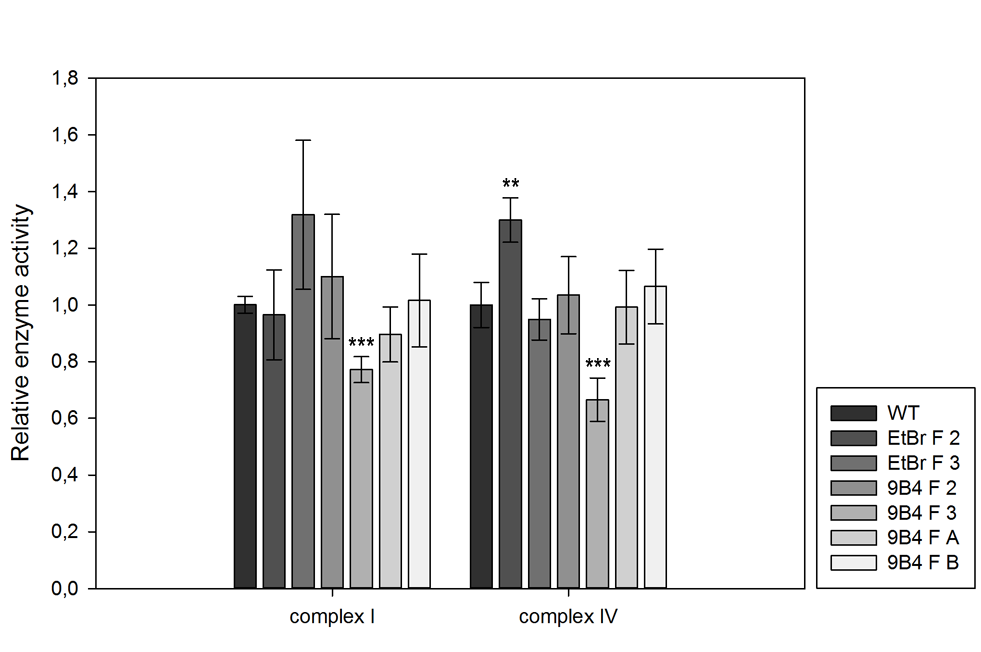

Supplement: Figure S2 — Relative activity of mitochondrial enzymes in PC-3 fusion cells. Enzyme activity of respiratory complex I and IV was measured spectrophotometrically in total cell lysates and was normalized to citrate synthase activity as reference activity. The data shown as ratio of wild type cells represent means ± SD from four independent experiments. *P<0.05, **P<0.01, ***P<0.001. Specific activity means of mitochondrial enzymes are shown in Table S1. (TIF) [file pone.0073207.s002.tif]
