# Supplementary material for: Efficient Repopulation of Genetically Derived Rho Zero Cells with Exogenous Mitochondria
Source: PLoS One. 2013 Sep 3;8(9):e73207. doi: 10.1371/journal.pone.0073207 (PMC3760891; doi:10.1371/journal.pone.0073207)
Supplement: Table S1 — Activity means of mitochondrial enzymes in PC-3 fusion cells. (DOC) [file pone.0073207.s003.doc]

**Table S1. Activity means of mitochondrial enzymes in PC-3 fusion cells.**

|  | Enzyme activity in nmol/mg/min | | |
| --- | --- | --- | --- |
|  | Citrate synthase | Complex I | Complex IV |
| PC-3 WT | 109.37 ± 2.59 | 10.55 ± 0.26 | 25.71 ± 1.68 |
| PC-3 EtBr F 2 | 106.35 ± 6.54 | 11.77 ± 1.49 | 32.49 ± 1.29 |
| PC-3 EtBr F 3 | 93.73 ± 8.08 | 11.82 ± 1.27 | 21.48 ± 3.34 |
| PC-3 9B4 F 2 | 77.16 ± 3.95 | 7.91 ± 1.48 | 18.60 ± 3.25 |
| PC-3 9B4 F 3 | 105.51 ± 15.00 | 7.35 ± 0.67 | 15.99 ± 1.42 |
| PC-3 9B4 F A | 109.59 ± 3.06 | 8.62 ± 0.76 | 25.52 ± 2.99 |
| PC-3 9B4 F B | 104.47 ± 6.40 | 10.34 ± 2.23 | 25.94 ± 4.61 |
